# Atorvastatin Therapy during the Peri-Infarct Period Attenuates Left Ventricular Dysfunction and Remodeling after Myocardial Infarction

**DOI:** 10.1371/journal.pone.0025320

**Published:** 2011-09-28

**Authors:** Xian-Liang Tang, Santosh K. Sanganalmath, Hiroshi Sato, Qiuli Bi, Greg Hunt, Robert J. Vincent, Yong Peng, Gregg Shirk, Buddhadeb Dawn, Roberto Bolli

**Affiliations:** 1 Division of Cardiovascular Medicine and Institute of Molecular Cardiology, University of Louisville, Louisville, Kentucky, United States of America; 2 Cardiovascular Research Institute, Kansas University Medical Center, Kansas City, Kansas, United States of America; Virginia Commonwealth University Medical Center, United States of America

## Abstract

Although statins impart a number of cardiovascular benefits, whether statin therapy during the peri-infarct period improves subsequent myocardial structure and function remains unclear. Thus, we evaluated the effects of atorvastatin on cardiac function, remodeling, fibrosis, and apoptosis after myocardial infarction (MI). Two groups of rats were subjected to permanent coronary occlusion. Group II (n = 14) received oral atorvastatin (10 mg/kg/d) daily for 3 wk before and 4 wk after MI, while group I (n = 12) received equivalent doses of vehicle. Infarct size (Masson's trichrome-stained sections) was similar in both groups. Compared with group I, echocardiographic left ventricular ejection fraction (LVEF) and fractional area change (FAC) were higher while LV end-diastolic volume (LVEDV) and LV end-systolic and end-diastolic diameters (LVESD and LVEDD) were lower in treated rats. Hemodynamically, atorvastatin-treated rats exhibited significantly higher dP/dt_max_, end-systolic elastance (Ees), and preload recruitable stroke work (PRSW) and lower LV end-diastolic pressure (LVEDP). Morphometrically, infarct wall thickness was greater in treated rats. The improvement of LV function by atorvastatin was associated with a decrease in hydroxyproline content and in the number of apoptotic cardiomyocyte nuclei. We conclude that atorvastatin therapy during the peri-infarct period significantly improves LV function and limits adverse LV remodeling following MI independent of a reduction in infarct size. These salubrious effects may be due in part to a decrease in myocardial fibrosis and apoptosis.

## Introduction

Myocardial infarction (MI) frequently causes left ventricular (LV) dilatation associated with myocyte hypertrophy and interstitial fibrosis of the noninfarcted myocardium. These changes in LV geometry, referred to as remodeling, contribute to the development of depressed cardiac performance [1]. Ventricular remodeling is further characterized by cardiac fibroblast proliferation and apoptotic cell death [2]. These pathological processes can lead to cardiac fibrosis as a result of disproportionate accumulation of extracellular matrix components, which impedes both contraction and relaxation and is arrhythmogenic [2], [3], [4]. Although available therapies delay the progressive downward course of heart failure, the prognosis of this syndrome remains bleak [5], [6]. Therefore, it is of critical importance to develop therapeutic strategies that will effectively inhibit the development and progression of LV remodeling and failure after MI.

The 3-hydroxy-3-methylglutaryl coenzyme A (HMGCoA) reductase inhibitors, statins, lower plasma cholesterol levels and have been associated with reduced morbidity and mortality in patients with coronary artery disease [7], [8]. It is presumed that statins may cause regression or stabilization of atherosclerotic plaques by lowering serum cholesterol levels [9]. However, the beneficial effects of statins on coronary artery disease are not limited to their ability to lower plasma cholesterol [7], but involve various pleiotropic effects on atherosclerosis including reduction of plaque thrombogenicity, inhibition of cellular proliferation and migration, anti-inflammatory effects, and improvement of endothelial function [10]. Despite the widespread clinical use of statins for hypercholesterolemia and prevention of coronary artery disease, data are lacking on the effects of statins on clinical outcome in heart failure secondary to MI. Although some clinical trials suggest that statins may attenuate remodeling and inflammation in patients with cardiomyopathy [11], [12], a recent randomized placebo-controlled study of high-dose statin in patients with systolic heart failure has shown no effect on ventricular remodeling [13]. Thus, the role of statins in heart failure due to MI remains controversial. Therefore, the purpose of this study was to determine whether administration of a statin during the peri-infarct period attenuates the progressive LV chamber dilatation and contractile dysfunction in a rat model of MI.

## Materials and Methods

### Ethics Statement

All studies were performed in accordance with the guidelines of the Animal Care and Use Committee of the University of Louisville (Ky) School of Medicine and with the *Guide for the Care and Use of Laboratory Animals* (Department of Health and Human Services, Publication No. [NIH] 86-23). The animal experiments were approved by the Institutional Animal Care and Use Committee (IACUC) of the University of Louisville (# 08137).

### Treatment and surgical protocols

Female Fischer 344 rats (age 10–12 wk; body wt, 175–200 g, Harlan Sprague-Dawley, Inc.) received oral atorvastatin (10 mg/kg/d by gavage, group II, n = 20) or an equivalent volume of vehicle (water, group I, n = 20) daily for 7 weeks starting 3 weeks before and continuing for 4 weeks after MI. To adjust the daily dose of the drug, the body weight was measured twice a week. The dose of atorvastatin used in this study is considered safe [14] and is similar to previously used doses shown to have cardioprotective effects in terms of limiting infarct size in rats [15],[16]. As statin use has been reported to have cardioprotective effects in patients when initiated prior to the onset of MI [17] and in the first few weeks after MI [18], we chose to treat the animals before and after the onset of MI to mimic the common clinical situation in which patients are treated with statins chronically, i.e., during the entire peri-infarct period. Three weeks into the treatment, rats were anesthetized with ketamine (37 mg/kg) and xylazine (5 mg/kg), intubated, and ventilated with a rodent respirator (Harvard Apparatus). Under sterile conditions, a left thoracotomy was performed in the fourth intercostal space, and a 5-0 Prolene suture tied around the left anterior descending coronary artery at 2–3 millimeters from its origin. The chest was closed with a 3-0 silk suture. The animals continued to receive the assigned treatment and were euthanized at 4 weeks after coronary artery ligation.

### Echocardiography

Serial echocardiographic studies were performed 4 days before surgery (baseline, BSL) and at 48 h and 4 wk after infarction under light anesthesia (pentobarbital, 25 mg/kg, i.p.) [19]. The anterior chest was shaved and rats were placed in the left lateral decubitus position. Body temperature was maintained between 36.9°C and 37.3°C. Echocardiographic images were obtained using a Philips HDI 5000 SonoCT ultrasound system equipped with a 12-5 MHz phased-array probe fitted with a 0.3 cm standoff and a 15-7 MHz broadband linear probe. The heart was imaged in the para-sternal short axis view at the level of the papillary muscles to obtain LV wall thickness and ejection fraction (EF), and in the para-sternal long axis view to measure LV end-systolic and end-diastolic volumes (LVESV and LVEDV). All measurements were averaged in three consecutive cardiac cycles and analyzed off-line by a single blinded observer using the ProSolv image analysis software. All calculations were derived using standard formulas. LV end-systolic and end-diastolic diameters (LVESD and LVEDD) were measured from M-mode tracings obtained at the mid-papillary level and analyzed according to modified American Society for Echocardiography standards (posterior wall leading-edge to leading-edge and anterior wall trailing-edge to trailing-edge) [20].

### Hemodynamics

Hemodynamic studies were performed at 4 wk after MI, just before euthanasia. Rats were anesthetized with ketamine (37 mg/kg) and xylazine (5 mg/kg), intubated, and mechanically ventilated. Anesthesia was maintained with 1% isoflurane and the core temperature kept at 37.0°C with a heating pad throughout the study. A 2F microtip pressure-volume (PV) catheter (SPR-869, Millar Instruments) was inserted into the right carotid artery and advanced into the LV cavity. The right jugular vein was cannulated for fluid administration. After 20 min of stabilization, the PV signals were recorded continuously with an ARIA PV conductance system (Millar Instruments) coupled with a Powerlab/4SP A/D converter (AD Instruments), stored, and displayed on a personal computer. PV relations were assessed by transiently compressing the inferior vena cava with a cotton swab. Parallel conductance from surrounding structures was calculated by injecting a small bolus of 15% NaCl through the jugular vein. LV end-diastolic pressure (LVEDP), dP/dt_max_, end-systolic elastance (Ees), and preload recruitable stroke work (PRSW) were calculated using the PVAN software program (Millar) [21].

### Morphometry and histology

After the hemodynamic measurements, a polyethylene catheter filled with phosphate buffer (0.2 M, pH 7.4) and heparin (100 IU/ml) was advanced to the ascending aorta via the right carotid artery. In rapid succession, the heart was arrested in diastole by injecting 1.0 ml of a mixture of cadmium chloride (100 mM)/potassium chloride (3 M) through the aortic catheter. The heart was then excised and perfused retrogradely with phosphate buffer for ∼3 min to flush out residual blood in the coronary circulation, followed by perfusion with 10% neutral buffered formalin solution for 15 min. Perfusion pressure was maintained between 60 and 80 mmHg while end-diastolic pressure was kept at 8 mmHg. After perfusion-fixation, the atria and right ventricle were dissected from the left ventricle. The LV weight was measured. The heart was cut into four transverse slices (∼2-mm thick), which were processed, embedded in paraffin, sectioned at 4-µm intervals, and stained with Masson's trichrome. Images were acquired digitally and analyzed using NIH ImageJ (1.37v). From the Masson's trichrome-stained images, morphometric parameters including LV chamber diameter and infarct wall thickness were measured in each section. All anatomical parameters were corrected according to a uniform sarcomere length [22].

### Myocardial hydroxyproline content assay

Hydroxyproline was assayed using a procedure described previously [23]. The apex of the left ventricle was lysed. The sample was centrifuged at 4,000 rpm for 10 min. The supernatant was mixed with fresh chloramine T for 10 min and then with Ehrlich's reagent at 75°C for 20 min. After samples were cooled, optical density was read at 560 nm with a spectrophotometer that was adjusted by a blank. The blank was prepared by the same procedure but without cardiac tissues in the reaction mixture. Hydroxyproline concentration, expressed as µg/mg of wet heart weight, was then calculated as previously described [23].

### Detection of cardiomyocyte apoptosis

Apoptosis was determined in heart sections with in situ hairpin-1 ligation (hairpin-1-biotin probe: 5′-GCGCTAGACCT*GGTCTAGCGCA-3′; T* represents biotinylated deoxythymidine [dT]) [24]. Briefly, fixed sections (4-µm) were first digested with Proteinase K (25 µg/ml) for 15 min, then incubated with 50 µl of T4 ligation buffer (T4 DNA ligase, 1 mM EDTA, 15% polyethylene glycol) and a biotin labeled hairpin-1 probe for 16 h, and then incubated with streptavidin-conjugated Texas-Red. A total of 2,000 cardiomyocyte nuclei were counted in each section after co-staining with hairpin-1.

### Statistical analysis

Data are reported as mean±SEM. Measurements were analyzed by ANOVA followed by unpaired Student t-tests with the Bonferroni correction. A value of *P*<0.05 was considered statistically significant. All statistical analyses were performed using the SPSS software (version 8, SPSS, Inc., Chicago, IL).

## Results

A total of 40 rats (20 in each group) were initially enrolled in the study. Eight rats in the vehicle-treated group (group I) and 6 in the atorvastatin-treated group (group II) died within the first 24 h after coronary ligation due to ventricular fibrillation. An additional 5 rats died after the initial 24 h during the 4-wk follow-up period (3 in the vehicle-treated and 2 in the atorvastatin-treated group), so that a total of 9 and 12 rats completed the protocol in groups I and II, respectively. There were no significant differences in mortality, body weight, heart weight, and heart/body weight ratio between the two groups of rats.

### Myocardial infarct size

**Figure 1:**
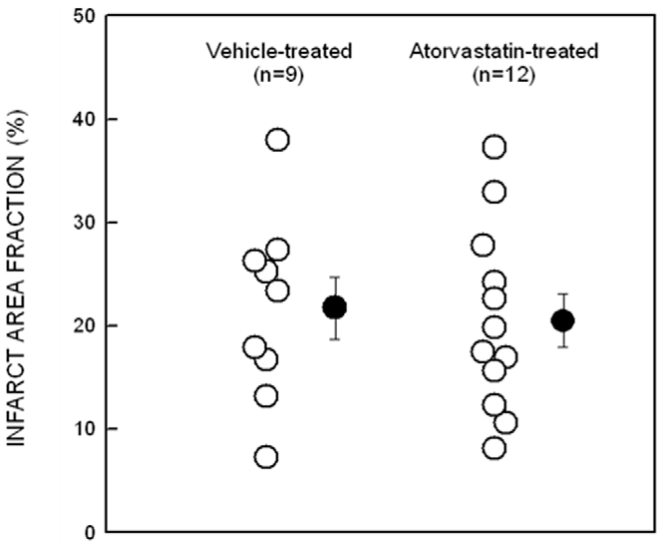
Myocardial infarct size. Myocardial infarct area fraction assessed from Masson's trichrome-stained hearts in vehicle-treated, and atorvastatin-treated rats 4 wk after coronary occlusion. ○, Individual rats; •, mean±SEM.

The infarct area fraction (which measures the average area of scarred tissue, expressed as a percent of the LV area in three LV sections 0.5–1.0 mm apart) was similar in atorvastatin- and vehicle-treated rats (20.5±2.7% vs. 21.7±3.2%, respectively), indicating that the extent of myocardial cell death produced by the coronary ligation was comparable in both groups (Fig. 1).

### Atorvastatin attenuates LV dysfunction

**Figure 2:**
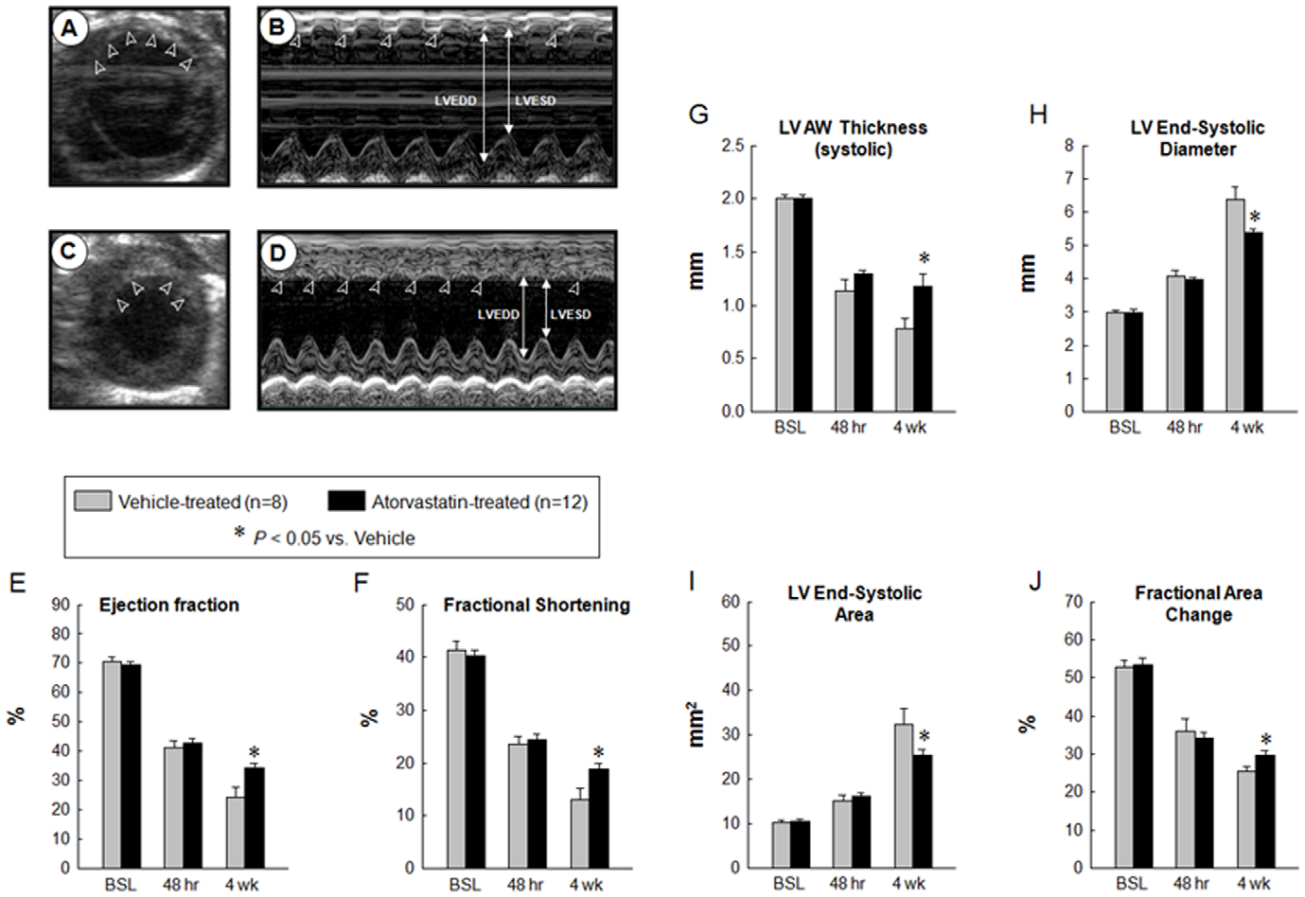
Echocardiographic assessment of LV function. Representative two-dimensional (A, C) and M-mode (B, D) images from vehicle-treated (A, B), and atorvastatin-treated (C, D) rats 4 wk after coronary occlusion. The infarct wall is delineated by arrowheads. Compared with the vehicle-treated heart, the atorvastatin-treated heart exhibited a smaller LV cavity, a thicker infarct wall, and improved motion of the infarct wall. Contractile activity in the infarct area is present in the atorvastatin-treated heart and virtually absent in the vehicle-treated heart. Panels (E–J) demonstrate that treatment with atorvastatin improved echocardiographic measurements of LV systolic function at 4 wk after myocardial infarction. Data are mean ± SEM. n = 8–12 rats per group. *, *P*<0.05 versus vehicle-treated rats at 4 wk. BSL, baseline.

At baseline (4 days before surgery), all parameters of LV function, measured by echocardiography, were similar in groups I and II (Fig. 2). At 48 h after surgery, the degree of LV systolic functional impairment did not differ among the groups (Figs. 2E–2J), indicating that the extent of injury sustained during MI was comparable. As expected, in vehicle-treated rats the infarct wall thickness decreased (Figs. 2A, 2B and 2G) and the LVESD increased at 4 wk of follow-up compared with baseline (Figs. 2A, 2B and 2H). In rats treated with atorvastatin, however, infarct wall thickness was greater (Figs. 2C, 2D and 2G) and the LVESD smaller (Figs. 2C, 2D and 2H) compared with vehicle-treated rats. Vehicle-treated rats exhibited a progressive deterioration in LVEF between 48 h and 4 wk after surgery; this worsening in LVEF was attenuated in rats treated with atorvastatin, resulting in a markedly greater LVEF at 4 wk compared with vehicle-treated rats (34.16±1.64% vs. 24.30±3.39%; P<0.05; Fig. 2E). Furthermore, LV systolic area was smaller and fractional area change was larger in atorvastatin-treated rats compared with vehicle-treated rats (Figs. 2I and 2J).

**Figure 3:**
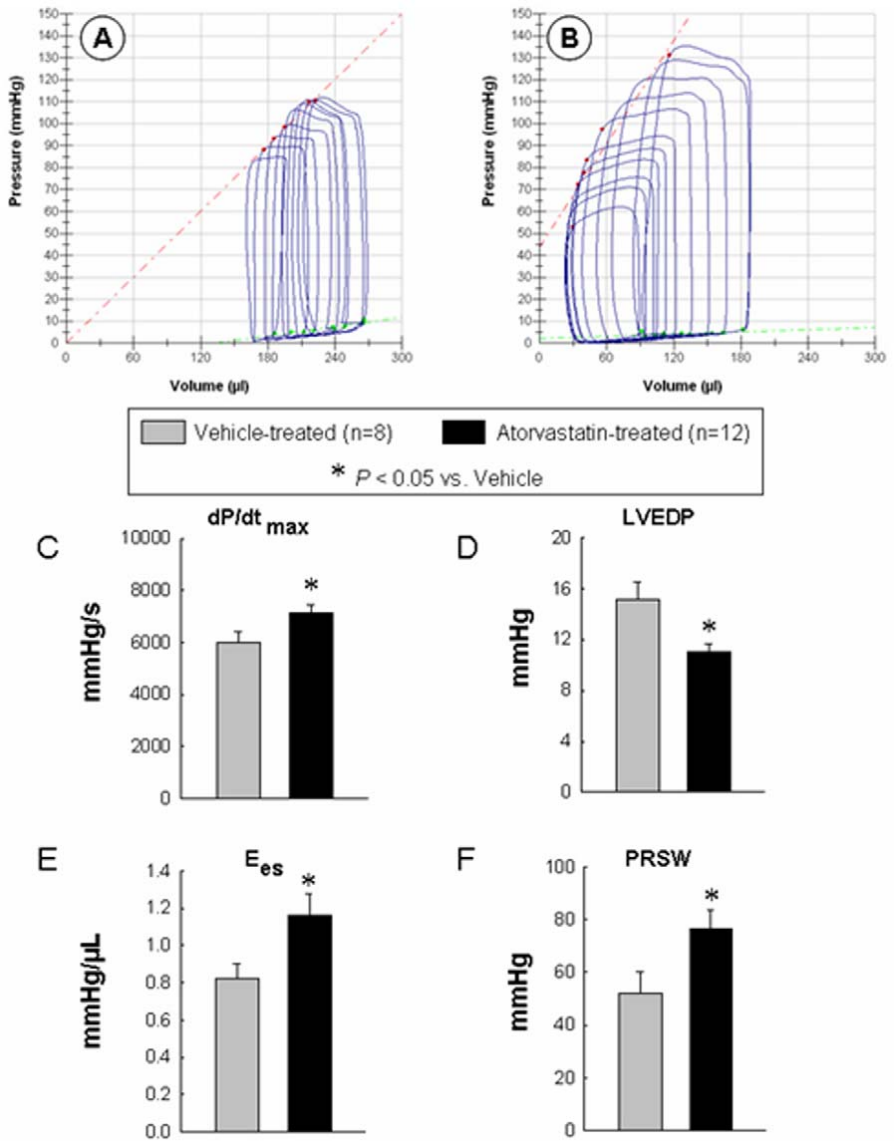
Hemodynamic assessment of LV function at 4 wk after atorvastatin treatment. Representative pressure-volume loops from a vehicle-treated (A) and an atorvastatin-treated (B) rat recorded during preload manipulation by a brief period of inferior vena cava occlusion. Panels C–F illustrate the quantitative analysis of hemodynamic variables including dP/dt (C), LV end-diastolic pressure (D), end-systolic elastance (E), and preload recruitable stroke work (F). Data are mean ± SEM. n = 8–12 rats per group. *, *P*<0.05 versus vehicle-treated rats at 4 wk. Abbreviations: Ees, end-systolic elastance; LVEDP, left ventricular end-diastolic pressure; PRSW, preload recruitable stroke work.

Consistent with the echocardiographic data, invasive assessment of cardiac function using the conductance catheter showed improved LV function in atorvastatin-treated animals. Fig. 3 shows representative P-V loops, dP/dtmax, LVEDP, end-systolic elastance (Ees), and preload recruitable stroke work (PRSW) obtained during IVC occlusion. Both load-dependent (LV dP/dt_max_, LVEDP) and load-independent (Ees, PRSW) parameters of LV performance were improved in the atorvastatin-treated group.

### Atorvastatin halts LV remodeling

**Figure 4:**
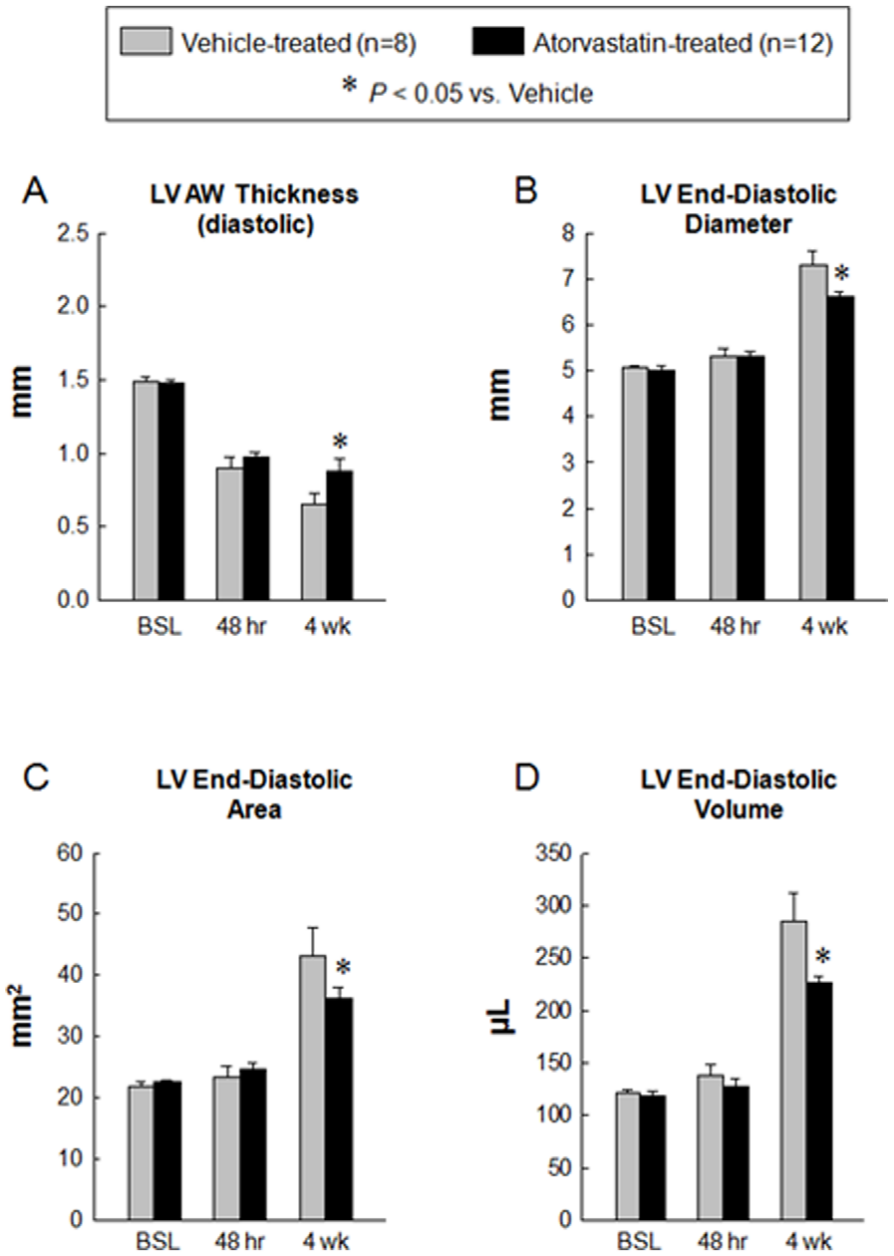
Quantitative assessment of LV remodeling. Panels A–D illustrates echocardiographic measurement of LV AW thickness in diastole (A), LV end-diastolic diameter (B), LV end-diastolic area (C) and LV end-diastolic volume (D) at baseline, 48 hr and 4 wk after myocardial infarction. The administration of atorvastatin improved adverse LV remodeling at 4 wk after myocardial infarction. Data are mean ± SEM. n = 8–12 rats per group. *, *P*<0.05 versus vehicle-treated rats at 4 wk. Abbreviations: AW, anterior wall; BSL, baseline; hr, hours; LV, left ventricular.

At 4 wk after MI, echocardiographic assessment showed an increase in LVEDD, LV end-diastolic area, and LVEDV in vehicle-treated rats, consistent with postinfarction LV remodeling (Figs. 2A, 2B and 4B–4D). However, in atorvastatin-treated rats, these variables were significantly smaller compared with vehicle-treated rats, indicating improvement in LV remodeling (Figs. 2C, 2D and 4B–4D).

**Figure 5:**
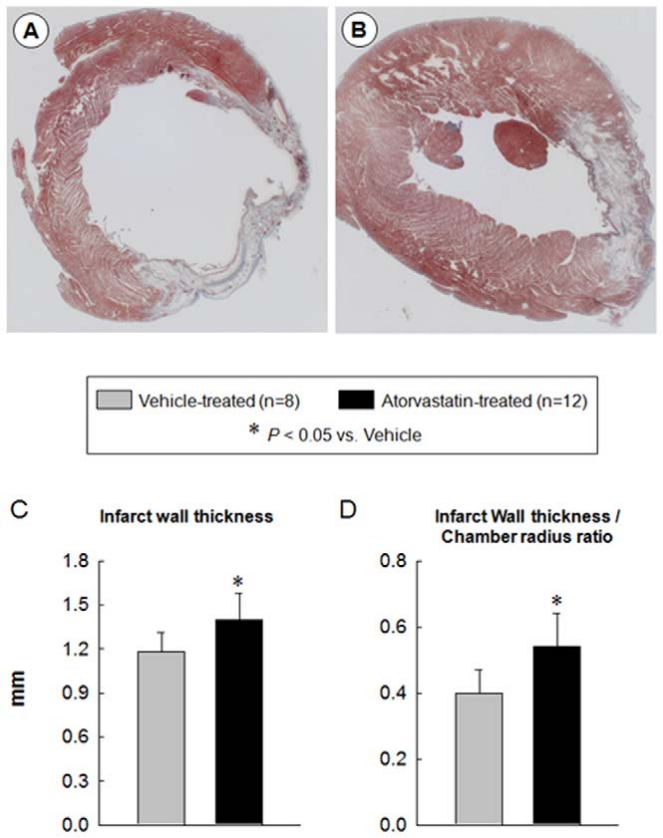
Morphometric assessment of LV remodeling. Representative Masson's trichrome-stained myocardial sections from vehicle-treated (A) and atorvastatin-treated (B) hearts. Scar tissue and viable myocardium are identified in white and red, respectively. Note that the LV cavity is smaller and the infarct wall thicker in the atorvastatin-treated heart. Panels (C, D) illustrate morphometric measurements of LV structural parameters. Data are mean ± SEM. n = 8–12 rats per group. *, *P*<0.05 versus vehicle-treated rats at 4 wk.

Morphometric analysis confirmed the echocardiographic findings. Compared with the vehicle-treated group, the LV chamber diameter was smaller and the infarct wall thickness and infarct wall thickness/chamber diameter ratio were greater in the atorvastatin-treated group (Fig. 5).

### Atorvastatin reduces hydroxyproline content and apoptotic cardiomyocytes

**Figure 6:**
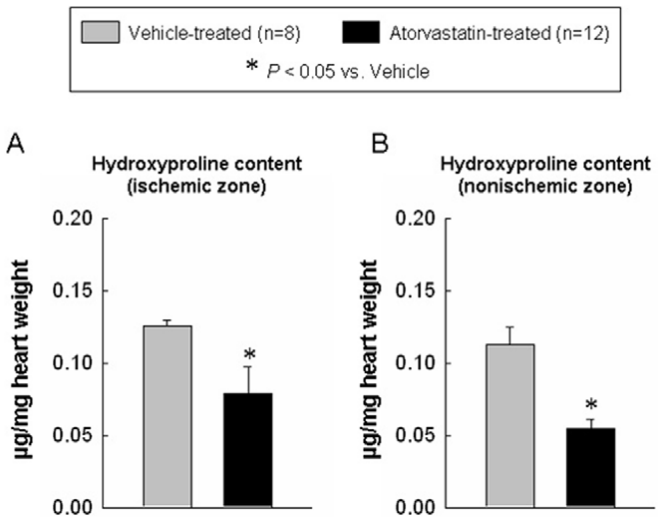
Myocardial collagen content. Panels A–B shows the quantitative assessment of collagen content in the ischemic zone (A) and non-ischemic zone (B) at 4 wk after myocardial infarction. The administration of atorvastatin decreased the collagen content at 4 wk after myocardial infarction. Data are mean ± SEM. n = 8–12 rats per group. *, *P*<0.05 versus vehicle-treated rats at 4 wk.

**Figure 7:**
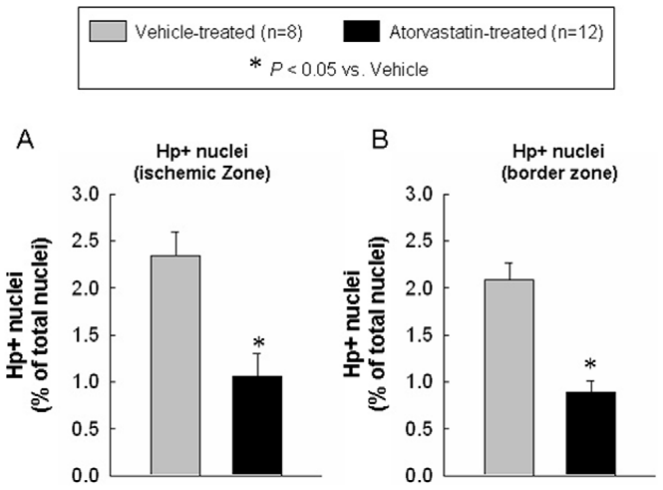
Quantitative assessment of apoptotic cardiomyocyte nuclei following in-situ ligation of hairpin oligonucleotides at 4 wk after myocardial infarction. Panels A and B show the quantitative assessment of apoptotic cardiomyocyte nuclei in the ischemic zone (A) and border zone (B). The administration of atorvastatin decreased the number of apoptotic nuclei at 4 wk after myocardial infarction. Hp+, hairpin positive. Data are mean ± SEM. n = 8–12 rats per group. *, *P*<0.05 versus vehicle-treated rats at 4 wk. The number of apoptotic cardiomyocytes was expressed as percent of total number of cardiomyocytes.

Because myocardial fibrosis contributes to the pathology of ventricular remodeling [25], [26], we examined whether atorvastatin attenuated myocardial collagen content. As shown in Fig. 6, the myocardial hydroxyproline content in the ischemic and nonischemic zones was significantly higher in the vehicle-treated group compared with the atorvastatin-treated group (0.13±0.01 vs. 0.09±0.02 and 0.11±0.01 vs. 0.05±0.01 µg/mg wet weight, P<0.05, respectively). In addition, myocardial apoptotic nuclear density (i.e., the percentage of nuclei that were apoptotic) was significantly higher in vehicle-treated compared with atorvastatin-treated hearts (ischemic zone: 2.35±0.25% vs. 1.06±0.24%; border zone: 2.09±0.18% vs. 0.89±0.13%, P<0.05) (Figs. 7A and 7B). Thus, atorvastatin treatment was associated with a lower percentage of apoptotic nuclei in both the ischemic and the border zones.

## Discussion

The major finding of this study is that administration of atorvastatin during the peri-infarct period improves LV function and attenuates adverse LV remodeling after MI independent of a reduction in infarct size. This salubrious effect was associated with a decrease in myocardial fibrosis and in cardiomyocyte apoptosis.

Our observations of preserved LV function and mitigated LV remodeling are consistent with previous studies of statins in acute MI. In animal models of MI, cerivastatin (started on the 7^th^ day after MI) [27] and fluvastatin (administered for 4 weeks after MI) [28] have been reported to increase survival and improve cardiac function along with an attenuation of LV remodeling. Furthermore, results from clinical studies in patients with idiopathic dilated cardiomyopathy [29] and nonischemic forms of heart failure [11], [30] also suggest that statins may improve the clinical status of patients by improving LV function and attenuating adverse remodeling in heart failure. Similarly, statin therapy has been shown to reduce the extent of periprocedural non-Q-wave MI [31], microembolization and microinfarction [32] when started before percutaneous coronary intervention.

The precise mechanism(s) whereby atorvastatin improved LV function is unclear. The drug did not reduce infarct size (Fig. 1). In contrast, previous studies of statins in animal models of ischemia-reperfusion injury have shown reduction of infarct size and limitation of infarct expansion due to salvage of jeopardized myocardium [33], [34], [35]. The lack of early reperfusion in our experimental model is likely to account for the lack of effect of atorvastatin on infarct size, since salvage of ischemic myocardium is not possible when the coronary occlusion is permanent [36]. It is conceivable that the reduction in collagen deposition (Fig. 6) and apoptosis (Fig. 7) in the surviving myocardium resulted in less fibrosis and greater preservation of contractile elements, thereby contributing to the alleviation of LV dysfunction. It is unlikely that changes in loading conditions could account for the salubrious effects of atorvastatin because this drug has been reported to have negligible effects on arterial pressure and heart rate [14]. Among the many actions of statins that could contribute to the improvement of LV function and structure after MI are enhanced nitric oxide formation [37], inhibition of inflammation [38], and normalization of augmented sympathetic outflow [39]. In addition, by maintaining intracellular calcium homeostasis, atorvastatin might modulate the expression of sarcoplasmic reticulum calcium handling proteins and improve myocardial function [40].

The finding that atorvastatin therapy led to a substantial reduction of myocardial collagen content is novel. Although reduction in collagen content by atorvastatin has been demonstrated in animal models of diabetic cardiomyopathy [41] and in spontaneously hypertensive rats [42], such an effect of the drug has not been previously reported in the setting of MI. The end-diastolic pressure-volume relationship, which indicates diastolic stiffness, was improved by atorvastatin treatment. Unlike LVEDP, which is load dependent, diastolic stiffness is independent of load. Passive stiffness is a passive viscoelastic property that returns the myocardium to its resting state [43]. An increase in passive stiffness can be caused by abnormalities in cardiomyocytes, extracellular matrix, or both [44]. Our finding that LV fibrosis was decreased in the atorvastatin-treated group offers a plausible explanation for the decreased diastolic stiffness in these animals. Myocardial fibrosis (a major feature of LV remodeling after MI) is driven by angiotensin II [45], [46]. Since statins have been shown to prevent angiotensin II-induced hypertrophy in cultured neonatal rat cardiac myocytes (probably by attenuating angiotensin II-stimulated p21 ras activity [47]), it is conceivable that angiotensin II antagonism was an important mechanism whereby atorvastatin improved diastolic stiffness.

Hairpin positivity reflects DNA break points, characteristic of apoptosis [48]. The number of hairpin positive myocytes was lower in atorvastatin-treated compared with vehicle-treated hearts (Fig. 7). Whether this antiapoptotic effect of statins was due to direct protective actions or was secondary to improved LV function cannot be resolved from the present data.

Our data have obvious translational implications. They suggest that the use of atorvastatin during the peri-infarct period might be useful for limiting adverse LV remodeling and improving LV function after MI. This approach might have a potential use in clinical settings such as cardioprotection before surgical procedures, including cardiac and major non-cardiac vascular surgery, and patients with acute coronary syndromes. The exact mechanism(s) of this protective effect, the minimal effective dose, and the minimal duration of treatment required to achieve a beneficial effect need further elucidation.
